# Subclinical Ventricular Dysfunction Detected by Speckle-Tracking Two Years after Use of Anthracycline

**DOI:** 10.5935/abc.20150102

**Published:** 2015-08

**Authors:** Aguinaldo Figueiredo Freitas Jr., Raquel Oliveira Santos, Salvador Rassi

**Affiliations:** 1 Serviço de Cardiologia da Faculdade de Medicina da Universidade Federal de Goiás, Goiânia, Goiás - Brazil

**Keywords:** Heart Failure, Ventricular Dysfunction Left/chemically induced, chocardiography, Anthracyclines/adverse effects

**Dear Editor**

We would like to congratulate the authors for the publication of their article "Subclinical Ventricular Dysfunction Detected by Speckle-Tracking Two Years after Use of Anthracycline", considering the great practical applicability of the theme. Cardiotoxicity secondary to chemotherapy drugs is a reality that imposes, on cardiologists and oncologists, the challenge of prevention and/or early detection of this complication, which has high morbidity and mortality^1^.

In this regard, we read with interest the abovementioned article, which highlights the usefulness of speckle-tracking to attain an early diagnosis of subclinical ventricular dysfunction, although this finding does not directly imply in implementing treatment due to the lack of current scientific evidence, which makes studies in this area even more important.

However, we would like to point out some aspects to add to the scientific information brought on by this article. In the Results section, the authors demonstrate that almost 80% of patients had also received cyclophosphamide as a chemotherapy drug, an alkylating agent that may be associated with ventricular dysfunction rates of up to 25% of the cases^2,3^, which cannot be minimized in the Discussion and Conclusion sections of this study. This same rationale can be applied to the more than 50% of patients receiving radiotherapy in the mediastinal region, regardless of the treated hemithorax, since the incidence of coronary heart disease in these patients is a side effect of significant incidence^4^.

We also observed a high rate of hypertension in both groups of patients and controls and that the systolic and diastolic BP levels were higher in the latter than in the first group. We would like to know if there was any difference between the groups regarding the class of antihypertensive drug used, as data in the literature suggest some protective effect of ACE inhibitors and beta-blockers on the incidence of ventricular dysfunction and major clinical outcomes^5^.
