# Supplementary material for: Subclinical Ventricular Dysfunction Detected by Speckle-Tracking Two Years after Use of Anthracycline
Source: Arq Bras Cardiol. 2015 Aug;105(2):206–7. doi: 10.5935/abc.20150102 (PMC4559134; doi:10.5935/abc.20150102)
Supplement: Related texts

# Reply

**Authors:** André L C Almeida, Edval Gomes dos Santos Júnior

**Dear authors**

We appreciate your interest and comments about our article.

Cyclophosphamide-associated cardiotoxicity presents as a syndrome of heart failure, myocarditis, pericarditis, or their association and can lead to death. It has an acute onset, with signs and symptoms occurring within one to ten days after the first dose, lasting for approximately one week^1^. Delayed cardiotoxicity development (> 3 weeks) is very rare in patients that survive the initial event^2-4^. In our sample, patients were assessed after a median of two years after the end of chemotherapy, making it unlikely that the obtained results can be attributed to the use of cyclophosphamide.

The observation about the increased incidence of coronary artery disease (CAD), as a side effect of radiotherapy in patients with breast cancer is absolutely pertinent. This increase is proportional to the number of cardiovascular risk factors the patients have and the mean dose of radiation to the heart. Previous data demonstrated that CAD development after radiation therapy occurred after a longer period of follow-up: 82 months, on average^5^.

Recently, Darby et al6 showed an increase of 16.3% (per Gray of radiation) in the rate of major coronary events in the first four years after radiation therapy in women with breast cancer6. This increase begins in the first five years after radiotherapy and persists for at least 20 years^6^. The development of new technologies in the field of radiation therapy has shown to be favorable to reduce this side effect^7^. In our study, however, none of the patients had coronary event during the study period.

Although there was no difference in the percentage of participants considered hypertensive in both groups (p = 0.71), the controls had higher levels of systolic and diastolic BP at the time of evaluation. As stated in the Discussion section, we attribute this fact to greater adherence to antihypertensive therapy among patients who were undergoing more stringent medical follow-up in the post-chemotherapy period. The increase in blood pressure levels tends to compromise strain values^8^. However, despite these higher blood pressure levels in the control group, the strain values were more compromised in the group using doxorubicin (DOX), which reinforces the importance of this drug as an independent predictor of reduced εLL and εCC in our patients.

There was no significant difference between the groups regarding the class of antihypertensive drug used: fourteen patients (34%) from the control group used angiotensin‑converting enzyme inhibitors or Angiotensin II‑receptor blockers vs. ten patients (25%) in the group treated with DOX (p = 0.367). One (2.4%) participant from the control group used a beta-blocker vs. five (12.5%) in the DOX group (p = 0.109).

## References

[1] Ayash LJ, Wright JE, Tretyakov O, Gonin R, Elias A, Wheeler C (1992). Cyclophosphamide pharmacokinetics: correlation with cardiac
toxicity and tumor response. J Clin Oncol.

[2] Gottdiener JS, Appelbaum FR, Ferrans VJ, Deisseroth A, Ziegler J (1981). Cardiotoxicity associated with high dose cyclophosphamide
therapy. Arch Intern Med.

[3] Braverman AC, Antin JH, Plappert MT, Cook EF, Lee RT (1991). Cyclophosphamide cardiotoxicity in bone marrow transplantation: a
prospective evaluation of new dosing regimens. J Clin Oncol.

[4] Pai VB, Nahata MC (2000). Cardiotoxicity of chemotherapeutic agents - incidence, treatment
and prevention. Drug Saf.

[5] Kalil R, Hajjar LA, Bacal F, Hoff PM, Diz M del P, Galas FR, Sociedade Brasileira de Cardiologia (2011). I Brazilian guideline for cardio-oncology from Sociedade Brasileira
de Cardiologia. Arq Bras Cardiol.

[6] Darby SC, Ewertz M, McGale P, Bennet AM, Blom-Goldman, Bronnum D (2013). Risk of ischemic heart disease in women after radiotherapy for
breast cancer. N Engl J Med.

[7] Travis LB, Ng AK, Allan JM, Pui CH, Kennedy AR, Xu XG (2012). Second malignant neoplasms and cardiovascular disease following
radiotherapy. J Natl Cancer Inst.

[8] Marwick TH, Leano RL, Brown J, Sun JP, Hoffmann R, Lysyansky P (2009). Myocardial strain measurement with 2-dimensional speckle-tracking
echocardiography: definition of normal range. JACC Cardiovasc Imaging.
